# Bioprocess Development for Lantibiotic Ruminococcin-A Production in *Escherichia coli* and Kinetic Insights Into LanM Enzymes Catalysis

**DOI:** 10.3389/fmicb.2019.02133

**Published:** 2019-09-13

**Authors:** Elvis L. Ongey, Lara Santolin, Saskia Waldburger, Lorenz Adrian, Sebastian L. Riedel, Peter Neubauer

**Affiliations:** ^1^Bioprocess Engineering, Institute of Biotechnology, Technische Universität Berlin, Berlin, Germany; ^2^Department of Isotope Biogeochemistry, Helmholtz Centre for Environmental Research, Leipzig, Germany; ^3^Chair of Geobiotechnology, Institute of Biotechnology, Technische Universität Berlin, Berlin, Germany

**Keywords:** lantibiotic, ruminococcin-A, bioprocess development, recombinant protein production, *Escherichia coli*

## Abstract

Ruminococcin-A (RumA) is a peptide antibiotic with post-translational modifications including thioether cross-links formed from non-canonical amino acids, called lanthionines, synthesized by a dedicated lanthionine-generating enzyme RumM. RumA is naturally produced by *Ruminococcus gnavus*, which is part of the normal bacterial flora in the human gut. High activity of RumA against pathogenic *Clostridia* has been reported, thus allowing potential exploitation of RumA for clinical applications. However, purifying RumA from *R. gnavus* is challenging due to low production yields (<1 μg L^–1^) and difficulties to cultivate the obligately anaerobic organism. We recently reported the reconstruction of the RumA biosynthesis machinery in *Escherichia coli* where the fully modified and active peptide was expressed as a fusion protein together with GFP. In the current study we developed a scale-up strategy for the biotechnologically relevant heterologous production of RumA, aimed at overproducing the peptide under conditions comparable with those in industrial production settings. To this end, glucose-limited fed-batch cultivation was used. Firstly, parallel cultivations were performed in 24-microwell plates using the enzyme-based automated glucose-delivery cultivation system EnPresso^®^ B to determine optimal conditions for IPTG induction. We combined the bioprocess development with ESI-MS and tandem ESI-MS to monitor modification of the precursor peptide (preRumA) during bioreactor cultivation. Dehydration of threonine and serine residues in the core peptide, catalyzed by RumM, occurs within 1 h after IPTG induction while formation of thioether cross-bridges occur around 2.5 h after induction. Our data also supplies important information on modification kinetics especially with respect to the fluctuations observed in the various dehydrated precursor peptide versions or intermediates produced at different time points during bioreactor cultivation. Overall, protein yields obtained from the bioreactor cultivations were >120 mg L^–1^ for the chimeric construct and >150 mg L^–1^ for RumM. The correlation observed between microscale and lab-scale bioreactor cultivations suggests that the process is robust and realistically applicable to industrial-scale conditions.

## Introduction

The accelerated growth of resistant pathogenic bacteria against the existing battery of anti-infective agents has prompted efforts in developing alternative drug candidates that possess multiple mechanisms of action to counter the engrossing effect of disease burdens. Lantibiotics possess characteristics that are very attractive to this research area and therefore, they are highly considered as alternative drug candidates to fight bacterial resistances (Cotter et al., 2013; Dischinger et al., 2014). However, their application in medicine is limited by low product yields and cultivation difficulties that is usually associated with the use of native producers to purify the desired compounds. That is the reason why despite the urgent needs for potent anti-infective agents, only few companies have invested little efforts in developing them, as well as presumed low profits (Servick, 2015). In a recent report, we assessed the chemical synthetic methods and biological means as production routes for lanthipeptides and arrived at the conclusion that *in vivo* production in microbial cells is a realistic alternative to attain industrially viable quantities (Ongey and Neubauer, 2016).

**FIGURE 1:**
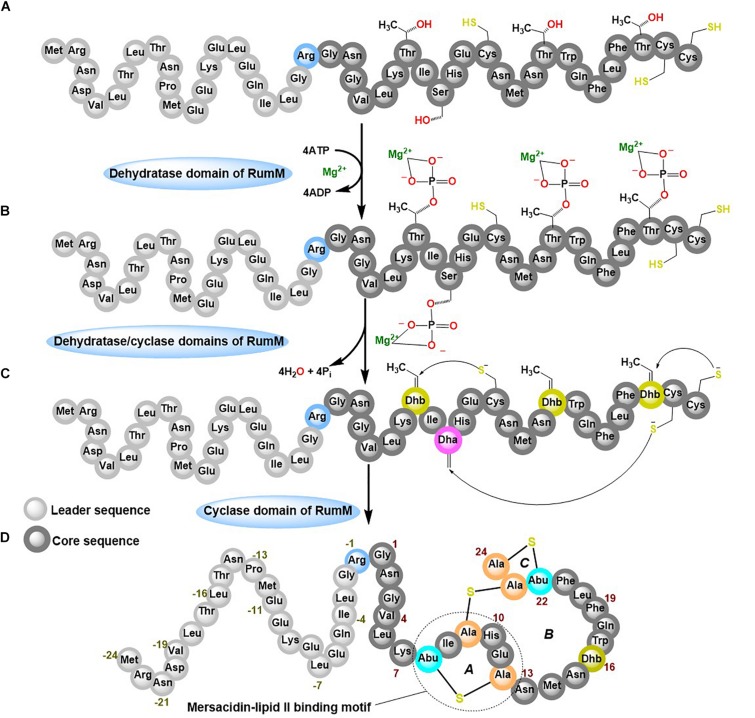
Post-translational modifications (PTMs) of ruminococcin-A by RumM. The dehydratase domain of the RumM enzyme converts serine and threonine residues in the core peptide of preRumA **(A)** into a phosphorylated intermediate **(B)** which subsequently undergoes an elimination reaction to yield a dehydro-intermediate **(C)**. The cyclase domain then conjugates the dehydroamino acid residues to cysteinyl thiols via a Michael-type addition cyclization reaction to yield the three-membered ring compound **(D)**.

Ruminococcin-A (RumA) is a class II lanthipeptide (also called lantibiotic because of its antibacterial activity) produced by the obligate anaerobe *Ruminococcus gnavus* E1. It was shown that the peptide can effectively kill pathogenic *Clostridia* (Dabard et al., 2001; Gomez et al., 2002), making it a potential candidate for disease treatment in humans and livestock, and perhaps it even may be utilized as preservative in the food industry. *R. gnavus* by its nature as an obligate anaerobic bacterium already poses substantial cultivation challenges that would certainly hinder the development of an efficient and robust production process. To overcome these limitations, we reconstructed the biosynthesis pathway of RumA in *Escherichia coli* and showed that all the desired post-translational modifications (PTMs) were correctly formed in the peptide resulting in a 3-membered ring compound shown in Figure 1D (Ongey et al., 2018). Two main genes are responsible for the biosynthesis and modifications of RumA namely; *rumA* which encodes the precursor peptide (preRumA) and *rumM* which encodes the dual-functional lanthionine synthetase (RumM). The leader peptide of preRumA (Figure 1A) serves as signaling moiety which RumM identifies and interacts with, to enable its activity on the core segment (Repka et al., 2017). The PTM process follows a series of events characterized by dehydration of threonine and serine residues in the core peptide into dehydrobutyrine (Dhb) and dehydroalanine (Dha), respectively. Michael-type addition cyclization reactions between the sulfhydryl group of cysteine and the resulting Dhb and Dha produce a lanthionine (Lan) or a methyllanthionine (MeLan) cross-bridge respectively (Schnell et al., 1988; Arnison et al., 2013; Yang and van der Donk, 2015; Repka et al., 2017). We applied this knowledge in developing the heterologous biosynthesis route. Moderate amounts of modified RumA precursor (6 mg L^–1^) was obtained with complex media cultivations in shake flasks (Ongey et al., 2018).

Class II lanthionine synthetases like the RumM are generally called LanM enzymes. They are bifunctional, possessing an N-terminal dehydratase domain and a C-terminal cyclase domain (Knerr and van der Donk, 2012). *In vitro* characterization of some LanM enzymes have revealed the use of ATP to phosphorylate serine or threonine, and ADP/Mg^2+^ as cofactors to eliminate the phosphate esters to yield Dha and Dhb (Xie et al., 2004; Bierbaum and Sahl, 2009; Ma et al., 2014; Shimafuji et al., 2015; Repka et al., 2017). Tandem mass spectrometry assays revealed that phosphorylation occurs in a unidirectional N- to C-terminal catalytic mode (Lee et al., 2009). However, conflicting reports exist about the catalytic modes of the different lanthipeptides (Lubelski et al., 2009; Krawczyk et al., 2012). Studies elsewhere suggest that the modifying enzyme may require longer time to catalyze formation of PTMs (Thibodeaux et al., 2014, 2016; Repka et al., 2017), but the modification machineries of lanthipeptides are too complex, severely hindering quantification of kinetic events involving lanthionine synthetases. Mass spectrometry assays have a potential in this regard because of its efficiency in detecting and distinguishing peptide species that are relevant to determine the type of changes that occur during the dehydration and cyclization reactions. Electrospray ionization mass spectrometry (ESI-MS) has been previously employed to quantify the relative concentrations of intermediates of class II precursor peptide during an *in vitro* LanM enzyme catalysis (Thibodeaux et al., 2014), but such an approach have not been attempted for *in vivo* systems.

The challenges encountered when expressing lanthipeptides in heterologous systems indicate that achieving good expression levels of the essential genes may not be an antidote for low production yield, but also to keep the cells viably active during the long cultivation period is required to guarantee the formation of desired PTMs. Reports suggest that some factors that influence cultivation like pH, aeration and cultivation medium may as well-contribute to the yields of some lantibiotics (Mortvedt-Abildgaa et al., 1995; Liu et al., 2005; Pongtharangku and Demirci, 2007; Chaney et al., 2009; Jiang et al., 2015). Although all these studies were performed with the natural producers, it is conceivable to anticipate such scenarios also for recombinant production. Thus, applying fed-batch cultivation techniques in the context of consistent bioprocess development may allow, aside from high cell densities, increasing yields of lantibiotics by adjusting physical parameters and medium composition.

High through-put optimization using a convenient fed-batch type technique like EnBase^®^ (Šiurkus et al., 2010; Glazyrina et al., 2012; Krause et al., 2016) allows controlled *E. coli* cultivations using a variety of conditions in microwell plates, while still ensuring high cell density growth. Feeding rates in the enzyme-based glucose delivery cultivation system is controlled by the amount of enzyme added, which generates a quasi-linear increase of the cell density. The strength of this technique was demonstrated for the non-ribosomally synthesized peptide valinomycin wherein the two complex non-ribosomal peptide synthetases responsible for its biosynthesis were reconstituted in *E. coli* and the system was optimized to attain 10 mg L^–1^ yield under lab-scale conditions (Li et al., 2014) and more than 2 mg L^–1^ under mimicked heterogenous large-scale conditions (Li et al., 2015).

The primary aim of the current study was to use RumA as an example to establish a process by rationally selecting suitable conditions from microwell plate cultivations and transfer to a larger-scale (3.7-L bioreactor, KLF Bioengineering, Switzerland). This therefore underscores a reasonably consistent approach to develop a high density fed-batch bioprocess for production of antimicrobial peptides and the potential for attaining economic viability. The secondary objective was to use the bioreactor set-up as a tool to gain insights into the kinetics of preRumA modifications by RumM. Thus, the present work combines bioprocess development approaches and ESI-MS and tandem ESI-MS as plausible tools to realize *in vivo* kinetic studies of lanthipeptide biosynthesis. The data reported herein, to the best of our knowledge, is the first recombinant process development study for lanthipeptides production with *E. coli*, using lab-scale bioreactors.

## Materials and Methods

### Bacterial Strains Description

Wild-type *E. coli* W3110 strain was applied as the expression host. This strain was transformed with the bicistronic vector pLEO*grA^∗^M1* (see Supplementary Figure S1). This vector harbors the genes that encodes the ruminococcin-A precursor peptide (preRumA) as a fusion construct to the C-terminus of GFP, as well as the ruminococcin-A lanthionine synthetase (RumM). The transformed strain was named *E. coli* W3110 pLEO*grA^∗^M1* (Ongey et al., 2018). The GFP-preRumA fusion construct had a TEV cleavable site linking GFP to the precursor peptide and a single Gly/Arg mutation at position −1 of the preRumA sequence (Figure 1) to enable complete cleavage of the leader sequence by trypsin. The genes encoding the chimeric fusion construct (His6-GFP-TEV-preRumA) and RumM are arranged in an operon on the pLEO*grA^∗^M1* vector.

### Pre-experiments in 24 Deep-Well Plates

The EnPresso B^®^ growth system (Enpresso GmbH, Berlin, Germany) was applied for parallel cultivation of *E. coli* strain W3110 pLEO*grA^∗^M1* under different growth and induction conditions. Regular 24-square deep-well plates (Ritter, Schwabmünchen, Germany) were used for the pre-experiments. *E. coli* W3110 pLEO*grA^∗^M1* was first grown in 100 mL EnPresso B medium in a 500-mL Ultra Yield Flask^TM^ closed on top using 0.2 μm re-sealable and sterile AirOtop^TM^ membranes (both Thomson Instrument Company, United States), incubated in an orbital shaker (Infors HT, Switzerland, 5 cm amplitude) at 30°C, 250 rpm overnight, attaining an optical density (OD_600_) of around 7–8 after 14–16 h. Prior to induction, the 24 deep-well plate was prepared by adding the desired amounts of IPTG for induction of protein expression and glucoamylase which releases glucose into the medium (see Supplementary Figure S2). This generated 24 different experiments representing variation in feeding rates and IPTG concentrations. The same amount of chloramphenicol added at the beginning of the cultivation (34 μg mL^–1^) was also added to the main culture at this time and 3 mL of the shake flask culture were supplied to the different wells of the plates. Sterile Breathable Films (Starlab, Hamburg, Germany) were used to seal the plates and incubated in the orbital shaker under the same conditions as before. At the end of the cultivation, the final pH of each well was measured and the respective OD_600_ of the cultures were estimated by manually diluting 200 μl of the culture and measuring the optical density using an Ultraspec 3300 spectrophotometer (Amersham, Germany).

### Online GFP Fluorescence, DO, and pH Measurements in Microwell Plates

Information obtained from the pre-experiments allowed us to design new experiments to evaluate certain parameters that affect a production process. The same conditions described for the pre-experiments in the previous section were replicated in four different plates. The first plate was a sterile 24 shallow-well flat-bottom plate (Ritter GmbH, Schwabmünchen, Germany) that was used to measure online GFP fluorescence signals [expressed as relative fluorescent unit (RFU)] using a Synergy^TM^ Mx monochromator-based multimode microplate reader (Biotek). The auto-fluorescence of a *gfp*^–^ strain was also measured as a control. The second plate was a 24 shallow-well flat-bottom HydroDish^®^ (HD24) plate (PreSens Precision Sensing GmbH) that possessed a pH sensor at the bottom of each well, and the third 24 shallow-well flat-bottom OxoDish^®^ (OD24) plate, also from PreSens, was equipped with oxygen sensors at the bottom of each well. The fourth plate was a 24 deep-well flat-bottom HydroDish (HD24-DW) plate. The HydroDish and OxoDish plates are sterile single use microwell plates, possessing pre-calibrated optical pH and DO sensors, configured to respectively measure pH and dissolved oxygen concentrations (DO) *on-line*. Culture volume in each well of the deep-well plate was set at 3 mL while the microwell plates were maintained at 1 mL per well. Duetz System sandwich covers and cover clamps (Enzyscreen, Heemstede, Netherlands) were applied to circumvent unprecedented reduction of liquid levels via evaporation. The growth of cultures in the first row of the fourth plate were determined *at-line* by manually diluting 5 μl of the culture in a 1-cm cuvette and estimating the concentration of cells at 600 nm using an Ultraspec 3300 spectrophotometer (Amersham). At the end of the cultivation, 1 mL culture from each well of the regular 24 shallow-well flat-bottom plate were collected in 1.5-mL Eppendorf tubes and centrifuged in a Himac/CT15RE centrifuge (VWR, Leuven) for 1 min at 16,000 × *g* at 4°C. Cell pellets were stored at −20°C.

### Growth Media, Preculture, and Inoculation Conditions for the Fed-Batch Bioreactor Cultivations

For the bioreactor cultivations, the preculture was prepared in two steps. First, a 20-mL LB medium culture in a 125-mL Ultra Yield Flask with AirOtop membrane was inoculated from actively growing colonies from a fresh LB agar plate and incubated for 7 h. Secondly, 500 μL of the pre-seed culture was used to inoculate 50 mL EnPresso B medium, which was supplemented with 3 U L^–1^ glucoamylase for controlled, linear glucose release. The preculture was incubated for 18 h. For the precultures 250-mL single-use polycarbonate sensor flasks (SFS-HP5-PSt3, PreSens Precision Sensing GmbH, Germany), which allow *on-line* monitoring of pH, DO and biomass, were used. All precultures were supplemented with 34 μg mL^–1^ chloramphenicol and incubated at 30°C and 200 rpm.

The initial volume of the mineral salt medium (MSM) used for the bioreactor cultivations was 1.95 L and composed of: 14.6 g L^–1^ K_2_HPO_4_, 4.0 g L^–1^ NaH_2_PO_4_ × 2H_2_O, 2.0 g L^–1^ Na_2_SO_4_, 2.5 g L^–1^ (NH_4_)_2_SO_4_, 0.5 g L^–1^ NH_4_Cl, 1.0 g L^–1^ (NH_4_)_2_-H-citrate and 0.1 mL L^–1^ antifoam PPG2000 that were autoclaved *in situ* and then complemented with sterile 0.1 g L^–1^ thiamin (filtered through 0.2 μm PTFE filter), 2 mL of a 1M solutions of MgSO_4_ × 7H_2_O, 2 mL L^–1^ trace elements and 9.0 g L^–1^ glucose (added from pre-autoclaved stock solutions). The trace elements solution was prepared as a 500X stock containing 0.5 g L^–1^ CaCl_2_ × 2H_2_O, 0.18 g L^–1^ ZnSO_4_ × 7H_2_O, 0.1 g L^–1^ MnSO_4_ × H_2_O, 20.1 g L^–1^ Na_2_-EDTA, 16.7 g L^–1^ FeCl_3_ × 6H_2_O, 0.16 g L^–1^ CuSO_4_ × 5H_2_O, 0.18 g L^–1^ CoCl_2_ × 6H_2_O, 0.085 g L^–1^ Na_2_SeO_3_ × 5H_2_O, 0.14 g L^–1^ Na_2_MoO_4_, and 0.725 g L^–1^ Ni(NO_3_)_2_ × 6H_2_O.

The bioreactors were inoculated with the EnPresso B precultures, which were in the linear growth phase (OD_583_ of 9–12, Supplementary Figure S3) to initial OD_583_ of approximately 0.3.

### Bioreactor Cultivation Conditions

For fed-batch cultivations, a 3.7-L KLF benchtop bioreactor (KLF 2000, Bioengineering AG Switzerland) with a maximum working volume of 3.0 L was used.

The cultivation temperature was kept constant at 30°C and the pH maintained at 7 ± 0.05 through controlled addition of 25% (v/v) ammonia solution. Stirring was performed using two six-blade Rushton impellers. The initial stirring speed was set to 400 rpm, whereas the initial air flow rate was set to 0.05 vvm. Both values were alternatively manually increased stepwise to ensure enough oxygen supply to the culture (DO > 15%). The stirring speed was increased in 100 steps until the full capacity of the reactor was reached (1,450 rpm). The air flow rate was gradually increased up to about 4 vvm.

The fed-batch cultivations were performed with an initial batch phase until glucose was completely depleted, followed by feeding phase where the glucose feed solution (with 50% w/v of glucose) was added exponentially at a specific growth rate (μ*_*set*_*) of 0.35 h^–1^ (corresponding to approximately 70% of μ*_*max*_* in the batch phase) according to Eq. 1.

(1) $$F\,\left(t\right)=F_{0}\cdot e^{\mu_{s\,e\,t}\cdot t}$$

The initial feed rate (g h^–1^) was calculated according to Eq. 2, where *Y*_*x/s*_ the biomass/substrate yield (calculated from the batch phase), *S*_*i*_ the concentration of the feeding solution, and *X*_*0*_ and *_*V_0*_* the biomass concentration (calculated from a correlation between previous OD_583_ and cell dry weight (CDW) measured values) and bioreactor liquid volume at the end of the batch phase, respectively.

(2) $$F_{0}=\frac{\mu_{s\,e\,t}}{Y_{x/s}\cdot S_{i}}\,\left(t\,X_{0}\,V_{0}\right)$$

The culture was induced with 200 μM IPTG when a stirring speed of 1,300 rpm and aeration rate of 2 vvm were reached. The inducer was added to the culture broth as well as to the feeding solution. After induction, the feeding rate was kept constant and eventually decreased to avoid anaerobic growth. Induction was performed for 10 or 21 h as given in the text.

In order to avoid undesired magnesium limitations, 4 mL of 1M MgSO_4_ was added every time the OD_583_ showed an increment of 20. Foam formation was suppressed by addition of small amounts of antifoam 204 (Sigma, Germany) when needed (maximum amount of less than 1 mL during the cultivation).

### Sampling and Analytical Methods During the Bioreactor Cultivations

During the cultivation sampling was performed every 1.5 h. At every sampling point, the OD_583_ of the culture broth was measured *off-line* in duplicates, manually with a spectrophotometer (Ultraspec 3000, GE Healthcare, Winsted, CT, United States, OD_583_-Photometer) and additionally with the automated pipetting system Cedex Bio HT Analyzer^®^ (OD583-Cedex; Roche Diagnostics International AG, Switzerland) with the OD Bio HT test kit (Roche Diagnostics International AG, Switzerland). Also, 2 mL aliquots were sampled in pre-weighted 1.5-mL tubes and dried at 80°C after centrifugation (4°C, 6,500 × *g*, 10 min) for later CDW determination. In addition, the supernatant of the samples were analyzed with the Cedex Bio HT Analyzer to assess consumption of glucose, Mg^2+^, ${\text{PO}}_{4}^{3}$, NH_3_, and accumulation of acetate with the test kits: Glucose Bio HT, Magnesium Bio HT, Phosphate Bio HT, NH3 Bio HT, and Acetate V2 Bio HT (Roche Diagnostics International AG, Switzerland). For preRumA peptide purification, pelletized cells from 2 mL samples (4°C, 6,500 × *g*, 10 min) were stored at −20°C.

### Ruminococcin-A Extraction and Analysis

Cell pellets were resuspended in lysis buffer (20 mM Tris-HCl pH 8, 300 mM NaCl, 10 mM imidazole, 10% glycerol) and the cells were lysed by sonicating on ice using the UP200S sonicator (Hielscher Ultrasonics GmbH, Teltow, Germany, 1 mm sonotrode diameter) for 10 min with 30% duty cycle and 30 s on/off intervals. The lysates were centrifuged for 15 min at 16,000 × *g* at 4°C to remove cell debris. Clarified lysates were purified using His SpinTrap columns (GE Healthcare, United Kingdom) and all samples were eluted in 0.5 mL elution buffer (20 mM Tris-HCl pH 7.4, 300 mM NaCl, 500 mM imidazole). The eluents were dialyzed in dialysis buffer (50 mM Tris-HCl pH 8, 150 mM NaCl, 1 mM DTT, 0.5 mM EDTA) and protein concentrations were estimated using the NanoDrop ND-1000 Spectrophotometer (Peqlab Biotechnologie, Erlangen, Germany). Samples from each elution were processed for SDS-PAGE analyses. Fresh homemade TEV protease was added (at a ratio of 1:100 w/w) to the dialyzed protein samples and then incubated overnight at 4°C. TEV digestion yielded preRumA, which was then extracted and purified using C18-ZipTips (Merck, Darmstadt, Germany). ZipTips-processed samples were used for mass spectrometric analyses as described later.

### SDS-PAGE and Quantification of His6-RumM and His6-GFP-TEV-preRumA

His SpinTrap column-purified samples obtained after dialysis were analyzed using 10% standard SDS gels. Buffers and gel recipes were assembled following standard procedures. SDS loading buffer was added to the samples and heated for 5 min at 95°C. BSA samples, used as standards for quantification, were prepared in the same manner and loaded on the gels alongside tested samples. All gels were visualized by staining with Coomassie G-250. Gels were scanned using a Konica Minolta c554 series PCL scanner. The areas of the bands were estimated using ImageJ and then compared against the BSA standards to obtain approximate quantities in mg L^–1^.

### Mass-Spectrometric Analyses

Samples purified using ZipTips were dried using a Concentrator Plus vacuum (Eppendorf, Hamburg, Germany) and subsequently resolubilized in 50% HPLC-grade acetonitrile. Dissolved samples were directly injected into a nanoLC-coupled Thermo Orbitrap Fusion Mass Spectrometer (Thermo Fisher Scientific) with a nano electrospray ion source. The samples were introduced into a capillary which was then heated by applying a high voltage across it to produce ions that are separated and measured by the orbitrap mass analyzer. The five times charged precursor preRumA ion (m/z = 1089.72) identified in the mass spectrum (MS1) was selected and channeled to the ion-routing multipole where it was fragmented, processed in the C-trap and analyzed by the orbitrap mass analyzer (MS2). A detailed description of the MS procedure is given in a previous publication (Ongey et al., 2018).

## Results

### Well-Plate Cultivations

**FIGURE 2:**
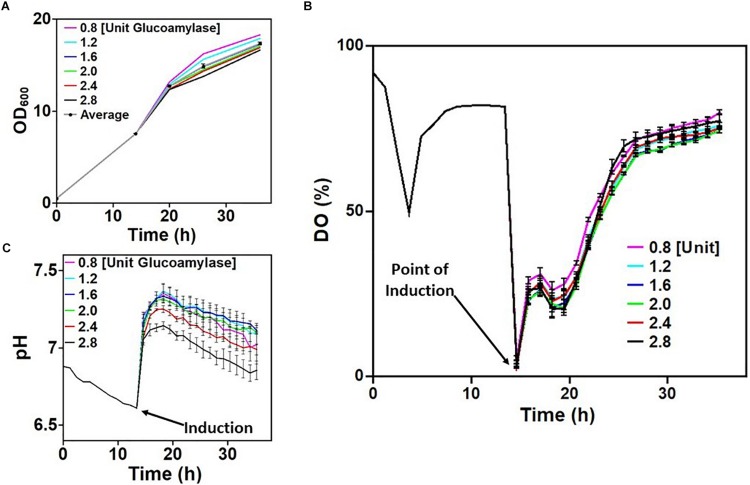
Screening cultivation-relevant parameters of *Escherichia coli* W3110 pLEO*grA^∗^M1*. **(A)** Growth (OD_600_) of cultures in the first row of the 24-microwell plates induced with 20 μM IPTG. **(B)** Dissolved oxygen concentration (DO) of cultures before and after boosting/inductions. **(C)** pH of cultures before and after boosting/induction. Time intervals from 0 to 14 h represent the culture in a shake flask. The curves for the DO and pH after induction are average values measured from all wells in each column of the 24 microwell plate. The plotted raw data is shown in Supplementary Figure S9.

Parallelized microwell plate cultivations were used to evaluate the behavior of *E. coli* W3110 pLEO*grA^∗^M1* under process-relevant conditions using *on-line* monitoring of DO and pH in EnPresso B cultures. Glucoamylase concentrations in the EnPresso B growth medium were varied from 0.8 to 2.4 U L^–1^ to determine the optimal glucose release rate. Before distribution to the 24-microwell plates the strain was first cultivated in a PreSens sensor shake flask. From the OD of the cultures measured, it was clear that the strain grew normally during cultivation (Figure 2A). Data recorded for the DO and pH during the first 14 h of cultivation were indicative of a typical *E. coli* growth profile (Figures 2B,C). Cells took up freely available glucose initially present in the EnPresso B medium during the first 5 h of cultivation and hence the growth profile exhibited characteristics of a typical batch cultivation, shown by the consistent drop in DO signals to about 40%. The rapid switch to glucose-limited growth was evident within 2 h after this drop, where the DO increased to 75% and remained stable at this level. Slow decrease in pH throughout the entire cultivation period was also observed (Figure 2C).

Induction of the initial culture was performed after 14 h of cultivation in 24-microwell plates with varying concentrations of IPTG and glucose feeding rates. Oxygen limitation could be detected at the start of plate measurements as all 24 cultures had initial DO values around 0%. This was due to lack of aeration since the culture was at a standstill during distribution into the wells. However, as aeration resumed, DO signals increased to about 25–40%, with slight variations which was dependent on the rate of glucose release in culture medium (Figure 2B). It is worth noting that growth was rapid between the period 14–20 h due to the addition of the booster tablet from the EnBase system, providing complex nutrients and by the additionally added biocatalyst (Figure 2A). Apparently, increasing the amount of glucoamylase (and consequently the rate of glucose release) had a minimum effect on the DO (Figure 2B). The booster tablet also led to an initial increase of the pH in dependence on the glucose release rate and a slower decline afterward (Figure 2C), so that all cultures remained at a pH above 6.6 until the end of the cultivation. Variations in pH observed across the culture wells in each column may be because all four wells in the column also received different concentrations of IPTG (refer to Supplementary Figure S2). The pH dropped with increasing rate of glucose release as shown in Figure 2C. In summary, all the cultures demonstrated a seemingly stable growth profile that mimicked a traditional fed-batch cultivation scenario.

**FIGURE 3:**
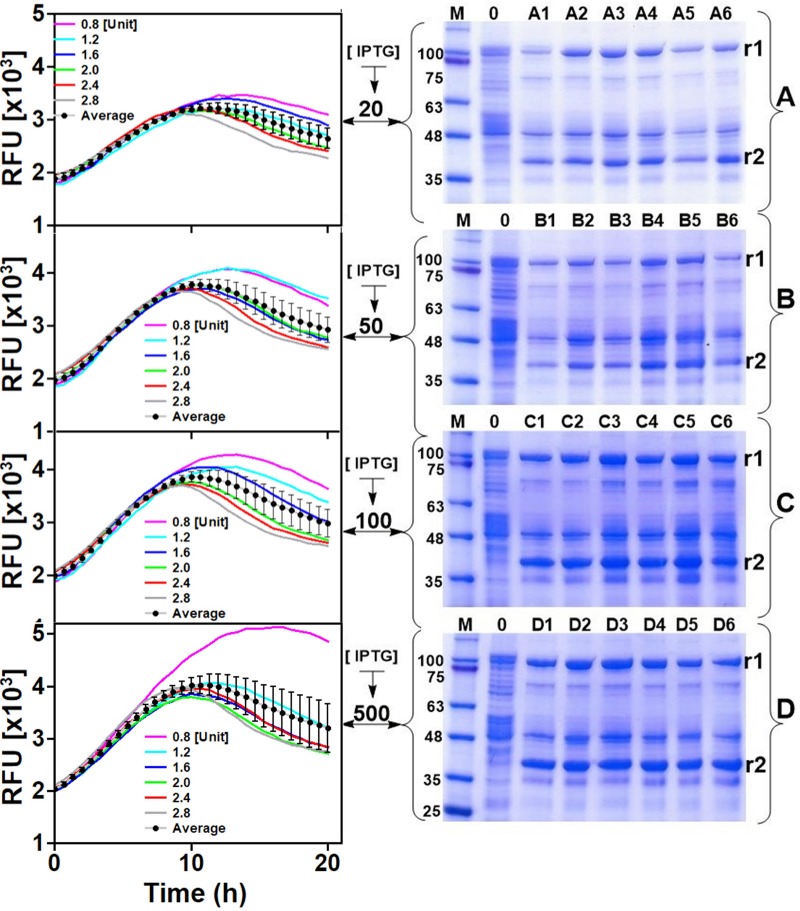
Expression optimization. The left panel indicates online GFP fluorescence measurements of *E. coli* W3110 pLEO*grA^∗^M1* cultivated in 24-microwell plates. **(A–D)** Represents rows of the 24-microwell plates while 1–6 represents the columns (see plate layout in Supplementary Figure S2). Each graph/gel therefore represents a row on the plate and the well numbers are indicated. Glucoamylase concentrations (in U L^–1^) added to wells 1–6 of each row are indicated in the graph. IPTG concentrations (μM) are also shown. The right panel shows the SDS-PAGE analyses of corresponding soluble His6-RumM (r1) and soluble His6-GFP-TEV-preRumA (r2) extracts obtained at the end of the cultivation. See Supplementary Figure S4 for full gel images. Average trendlines and error bars in the chart show the extend to which the fluorescence signals vary across the different cultures.

Examining the GFP fluorescence signals in the different cultures of the 24-microwell plate quickly gives the impression that product formation is negatively affected over long periods of cultivation. However, this may not necessary be the case as the SDS-PAGE analyses of HisTrap spin column-purified extracts from all the wells did not show the same trend. It could easily be identified that IPTG concentration influenced expression in all cases. For instance, navigating vertically through the wells of each column (e.g., column 1: A1→B1→C1→D1) as shown in the left panel of Figure 3, it is clearly visible that expression intensifies with increasing strength of induction. Conversely, increasing the rate of glucose release resulted in significant decreases in fluorescence signals approximately 10 h after induction. However, induction strength still had a positive influence on measured GFP fluorescence signals before this point. Averagely, there was significant variation in GFP signals across each row of the 24 microwell plate especially at higher induction strength. Analysis of the final product extracted from the wells indicated that excess glucose feeding did not have a positive impact on production (see wells A6, B6, C6, D6 in comparison to their counterparts in the same row), especially in cultures where IPTG concentrations were low. Meanwhile supplying excess glucose to the culture increased growth rate and consequently produced higher biomass yields, the same procedure is counterproductive with respect to target protein expression levels. Taking all these into account, IPTG concentrations in the range of 100–500 μM was considered suitable for effective cultivation of *E. coli* strain W3110 pLEO*grA^∗^M1* under glucose-limited conditions to ensure optimal production of His6-GFP-TEV-preRumA^∗^ and Hs6-RumM. Therefore, a high specific growth rate at the time of induction (70% of μmax) and an IPTG concentration of 200 μM were selected for the following bioreactor cultivations.

### Bioreactor Cultivations

**FIGURE 4:**
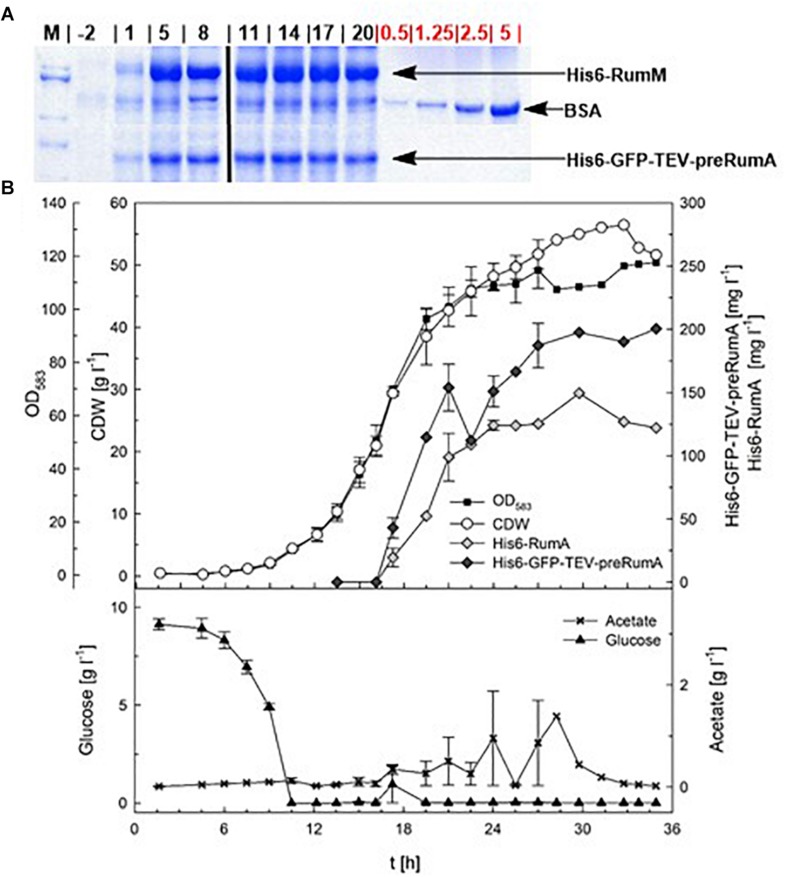
Fed-batch cultivation for RumA production with *E. coli* W3110 pLEO*grA^∗^M1.* **(A)** SDS-PAGE showing expression of His6-RumM and His6-GFP-TEV-preRumA from –2 to 20 h after induction. The amount of BSA (in μg, used as quantitative standard) were loaded is indicated on the gel. Full gel images are displayed in Supplementary Figure S8. **(B)** Cell dry weight (CDW, g L^–1^), OD_583_-Cedex, expression of His6-RumM (mg L^–1^) and His6-GFP-TEV-preRumA (mg L^–1^), glucose (g L^–1^) and acetate (g L^–1^) values are shown. Data points are the mean values obtained from duplicate cultivations and error bars indicate ± SD. Values after 27 h represent only one cultivation since the second cultivation was stopped 10 h after induction.

In order to evaluate the potential of *E. coli* W3110 pLEO*grA^∗^M1* for the biotechnological production of RumA, two fed-batch cultivations were performed in a 3.7-L lab-scale bioreactor. The strain had a maximum specific growth rate of 0.50 h^–1^ in the batch phase. The depletion of the initial glucose marking the end of the batch phase, could be observed via the sudden increase in the DO at ∼10 h (Figure 4B and Supplementary Figure S5). Exponential feeding was performed with 50% (w/v) glucose solution until induction with 200 μM IPTG (∼15 h), followed by constant feeding rate. During the fed-batch phase glucose concentrations in the bioreactor remained always limiting and acetate accumulation was controlled below 2 g L^–1^ by eventual reduction of the feeding rate (Supplementary Figure S6). A constant RQ value around 1 and a *k_*L*_a* value between 1,000 and 1,500 h^–1^ was reached during both cultivations, which showed a good reproducibility (Figure 4 and Supplementary Figure S7). RQ value of 1 means that *E. coli* respires aerobically in presence of the carbohydrate substrates, producing equal molar quantities of carbon dioxide as the molar amount of oxygen consumed.

Induction was performed for 10 or 21 h and at the end of the cultivation biomass concentrations of a maximum of 56.5 g L^–1^ CDW were achieved (Figure 4). Samples were obtained during the bioreactor cultivation at different time points, for preRumA analyses starting from 2 h before induction right up to 20 h after induction. His6-RumM and His6-GFP-TEV-preRumA were co-purified using His-tag spin columns. Results from SDS-PAGE analyses indicated strong expression of both constructs within 5 h after induction (Figure 4A). Product concentrations were estimated to be > 150 mg L^–1^ for His6-RumM and > 120 mg L^–1^ for His6-GFP-TEV-preRumA. The changes in production levels from 5 h after induction throughout the entire cultivation were not very significant (Figure 4B).

### *In vivo* Dehydration of preRumA by RumM

Data describing time-dependent changes in the concentrations of preRumA, partially modified intermediates thereof and RumA as the final product would be helpful in deciphering mechanisms of LanM enzymes and provide molecular details that may further identify other unknown features that assist in the process. Although overexpression of both, His6-GFP-TEV-preRumA and His6-RumM, were already significantly high within 5 h after induction, it was not possible to determine at this time whether the formation of the PTMs on preRumA were already achieved. Prior to the fed-batch bioreactor cultivations, it was assumed that ample time should be allowed for the PTMs to form, since other studies suggested that the modifying enzyme may require longer time to catalyze formation of PTMs (Thibodeaux et al., 2014; Repka et al., 2017). Taking these into consideration, we investigated modification of preRumA at different time points during the fed-batch bioreactor cultivations.

**FIGURE 5:**
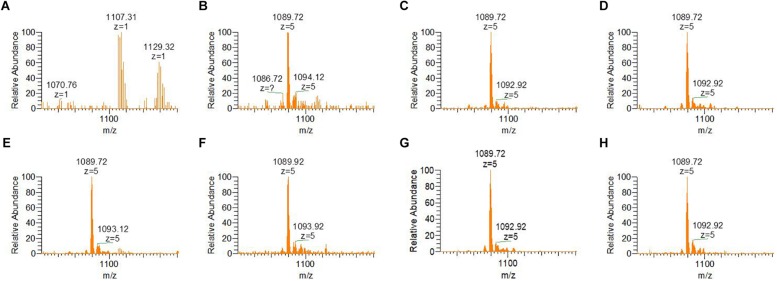
ESI-MS spectra indicating dehydration of preRumA from bioreactor cultivation of recombinant *E. coli* W3110 pLEO*grA^∗^M1*. **(A–H)** Represent samples obtained from –2 (negative control before induction) – 20 h post IPTG induction at an interval of 3–4 h. TEV-digested extracts of His6-GFP-TEV-preRumA were purified using ZipTips and directly injected into the mass spectrometer. The m/z_(z=__5__)_ = 1,089.72 peak represents tetra-dehydrated preRumA, while the minor peak represents the loss of three water molecules from preRumA. Molecular weight of non-dehydrated preRumA is 5,515.60 Da [m/z_(z=__5__)_ = 1,104.12].

Mass spectrometric analyses of preRumA cleaved from the chimeric His6-GFP-TEV-preRumA show that the precursor was fully dehydrated (or tetra-dehydrated) barely 1 h after IPTG induction, whereas as expected, for the extract obtained before induction, no peak was detected in the mass spectrum belonging to either tetra-dehydrated, partially dehydrated or non-dehydrated preRumA (Figure 5). Triple dehydrated preRumA [m/z_(z=__5__)_ = 1,092.92] was evident in all the samples from 1 h post-induction but the peak strength was very weak compared to the tetra-dehydrated version [m/z_(z=__5__)_ = 1,089.72].

**FIGURE 6:**
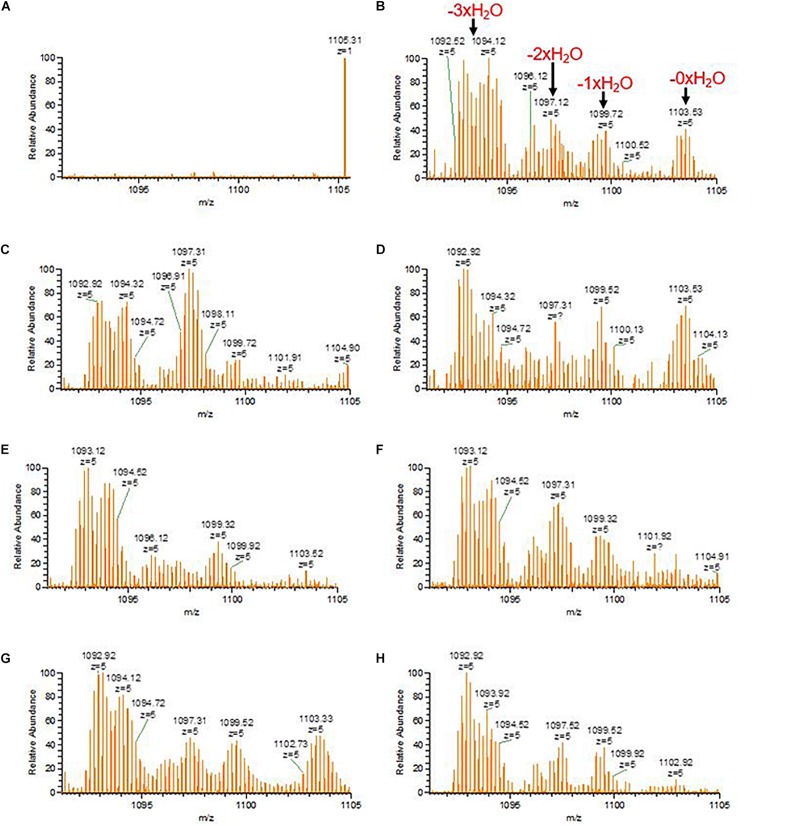
Fluctuations of incomplete preRumA dehydration products. **(A–H)** Represent samples obtained from –2 (negative control before induction) to 20 h post IPTG induction at an interval of 3 h. The products are: unmodified preRumA [m/z_(z=__5__)_ = 1,104.12], single dehydrated [m/z_(z=__5__)_ = 1,100.52], double dehydrated [m/z_(z=__5__)_ = 1,096.92], and triple dehydrated [m/z_(z=__5__)_ = 1,093.32]. Notice that the m/z value in the spectrum may represent a different isotope of the same product.

Considering the different intermediate products of preRumA, fluctuations were observed in all samples except for the negative control sample obtained 2 h before induction (Figure 6A). For instance, unmodified preRumA was apparent at 1 h post-induction (Figure 6B) but was almost disappearing at 5 h (Figure 6C) to accumulate again around 8 h post-induction (Figure 6D). Furthermore, in Figure 6D (8 h post-induction) and Figure 6G (17 h post-induction), the signal intensity of undehydrated preRumA is higher than the intensity at 1 h post-induction. Whereas it is almost undetectable at 11 h (Figure 6E), 14 h (Figure 6F), and 20 h (Figure 6H) after induction. The current data also shows that the changes in unmodified preRumA directly affect the single and double dehydrated components while the triple dehydrated version of preRumA remained overwhelmingly present throughout the cultivation. The fluctuations in the various intermediate components are expected since the fed-batch cultivation system may allow the expression of target proteins as the cells continue to receive more nutrients and grow, thereby providing a constant substrate pool for RumM.

### Lanthionine Formation in preRumA by RumM *in vivo*

The dehydratase function of RumM discussed in the previous section creates active centers for the cyclase domain to perform its function and since there is no difference in mass between the dehydrated peptide and the cyclized version, we could not detect ring formation from the ESI-MS data alone. Therefore, the precursor ion representing the tetra-dehydrated preRumA was isolated and channeled to the ion-routing multipole of the mass spectrometer for tandem MS measurements. The penta-charged peptide ion [m/z_(z=__5__)_ = 1,089.72] was fragmented using high-energy collisional dissociation to generate product ions having distinctive characteristics. In our previous report, we have already identified specific product ions that are produced as a result of the cross-linking in the core peptide (Ongey et al., 2018).

**FIGURE 7:**
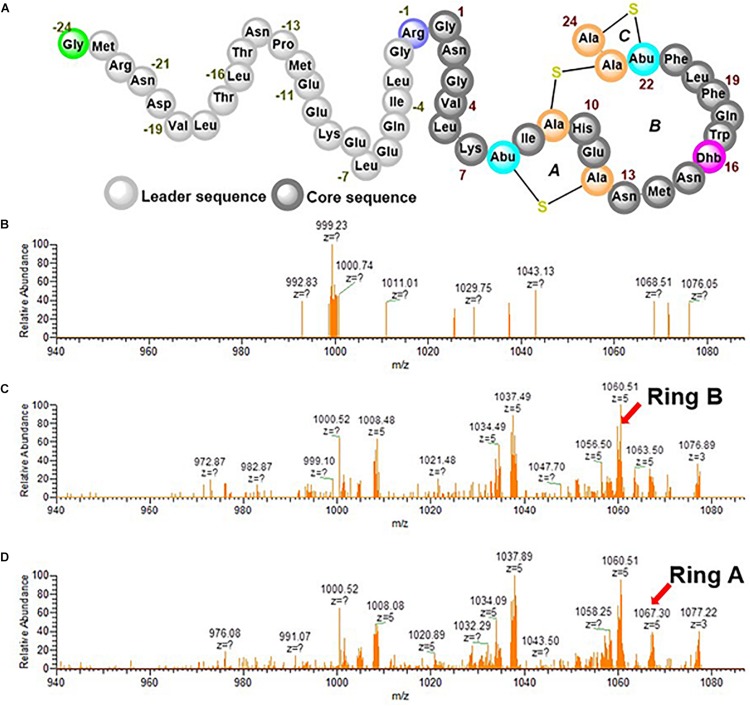
Tandem MS experiments of tetra-dehydrated preRumA [m/z_(z=__5__)_ = 1,089.72]. **(A)** Schematic structure of fully modified preRumA. The processed ESI-MS2 data displayed in the spectra were obtained at 1 h **(B)** 2.5 h **(C)** and 4 h **(D)** post IPTG induction. Intensive ion series representatives of Ring A and Ring B are indicated by arrows.

An internal ion [m/z_(z=__5__)_ = 1,067.30] is produced as a result of cleavage of peptide bonds linking both the N- and C-terminal sides of Ile8 in the core peptide of preRumA. This ion is characteristic of a cross-linkage between Thr7 and Cys12 (Ring A). Another important internal ion is produced by cleavage of peptide bonds linking both the N- and C-terminal sides of Phe21 [m/z_(z=__5__)_ = 1,060.51]. This represents Ring B which is formed via a cyclization reaction between Ser9 and Cys23. It was shown that Dhb16 is not engaged in any cross-linking reaction, leaving Dhb22 and Cys24 as the remaining residues which form Ring C (Dabard et al., 2001; Ongey et al., 2018). Other product ions shown in the spectra in Figure 7 represent different fragments resulting from preRumA fragmentation. The objective was to determine at what time these rings begin to form. Results show that there was no ring formation within 1 h of induction (Figure 7B), i.e., no product ion was identified representing either Ring A or Ring B. This therefore indicates that although the tetra-dehydrated preRumA was evidently present at 1 h post-induction, the cyclization reactions took a longer time to complete since fully functional thioether rings were identified from 2.5 h post-induction as shown in Figures 7C,D.

## Discussion

This study describes a bioprocess strategy to achieve biotechnological relevant production of lanthipeptides in high cell density bioreactor cultivations using ruminococcin-A as an example. The data reported herein were extracted from a combination of glucose-limited fed-batch cultivation methods, ESI-MS and tandem ESI-MS. Information derived from the data may be adapted to improve the biosynthesis pathway. These techniques can also be employed to quantify *in vivo* dehydroamino acids formation and lanthionine modifications catalyzed by lanthionine-generating enzymes during the production process in bioreactor cultivations.

The heterologous *E. coli* strain harboring the RumA biosynthesis machinery demonstrated stable growth and production characteristics during the two bioreactor cultivations, suggesting that the strain could also be processed under large-scale conditions with limited complications. The concentrations of His-tag purified His6-GFP-TEV-preRumA and His6-RumM obtained from the bioreactor cultivations were in the range of 120–200 mg L^–1^. Considering that preRumA is one sixth of the size of His6-GFP-TEV-preRumA, it can be estimated that 120 mg L^–1^ of the chimeric construct would yield > 10 mg L^–1^ of pure and functionally modified preRumA, which doubles the amount initially obtained from shake flask cultivation using complex media (Ongey et al., 2018). Additional studies to ascertain the influence of oscillating conditions on strain behavior and product quality during large-scale industrial bioreactor scenarios may be necessary to further evaluate the robustness of the strain.

However, the modification machinery of lanthipeptides is very complex, which hinders the establishment of standard quantitative approaches that can decipher the kinetic events of lanthionine synthetases. That is why the lab-scale fed-batch bioreactor cultivations were further exploited in conjunction with mass spectrometry to derive some information regarding the kinetics of preRumA modifications. The potential of mass spectrometry assays cannot be underestimated since they are capable of detecting and distinguishing peptide species that are relevant to determine the type of changes that occur during the dehydration and cyclization reactions (Thibodeaux et al., 2014). Tandem MS data shows that fully active preRumA with all the necessary thioether cross-bridges was apparent around 2.5 h post-induction. There was no evidence of cyclization at 1 h post-induction although the tetra-dehydrated preRumA was evidently present. These results are comparable to the modification rates obtained from *in vitro* studies involving class II lanthipeptide synthetases ProcM and HalM2 (Thibodeaux et al., 2014).

The fact that dehydration and cyclization occur at different time suggests that the dehydratase and cyclase reactions of LanM enzymes are independent of each other, corroborating an earlier report where they showed that the two reactions are largely independent for ProcM, with the dehydration reactions preceding cyclization (Mukherjee and Van Der Donk, 2014). This is in contrast with class I nisin cyclase which appears to install thioether rings before dehydration is completed, thereby generating partially dehydrated product due to inaccessibility of nisin dehydratase to target threonine or serine residue (Repka et al., 2017). However, this is conceivable since a single multi-domain enzyme is involved in class II peptide modifications while class I peptides are modified by two separate enzymes, one catalyzing dehydration and the other catalyzing cyclization (Repka et al., 2017). Based on the growth profile of the strain during the bioreactor cultivations and observations from SDS-PAGE and tandem MS data, we concluded that it would be reasonable to stop the bioreactor cultivation around 10 h post IPTG induction. That is why the second cultivation was discontinued at this point, which also happens to be the point where GFP fluorescence signals also began to decline during the microscale cultivations.

It may be challenging to provide a simple explanation for the constant accumulation of triple dehydrated preRumA. Since dehydration and cyclization reactions cannot be distinguished in time and space (Lubelski et al., 2009), the current data does not indicate which of the target threonine or serine residue in the core peptide of preRumA is unmodified in the triple-dehydrated version. Furthermore, tandem MS of the triple dehydrated component could not supply adequate fragmentation data to determine this because the amount of the component present in the sample was not enough. Additionally, there is no general rule regarding the directionality of catalysis involving lanthionine-generating enzymes because while some follow an N→C-terminal mode of catalysis (Lubelski et al., 2009), others prefer the C→N-terminal mode (Krawczyk et al., 2012). Therefore, it is not enough to just assume that the extreme C- or N-terminal threonine is the unmodified residue in this case. A more concentrated sample may be applied to enhance the signal strength of the triple-dehydrated component and allow generation of enough tandem MS data.

Initially, microscale cultivations involving the easy fed-batch technique that makes use of an enzyme-based automated glucose-delivery system in a mineral salt-based medium was applied to produce the peptide in *E. coli*. Microscale cultivation is an innovative strategy that facilitates bioprocess development by reducing experimental costs and allowing parallel assessment of different process relevant parameters. The development and application of the convenient EnBase technology (Krause et al., 2016) which allows a continuous controlled delivery of glucose to the culture, revolutionized this aspect by allowing consistent data extraction for these parameters that could be directly applied in large-scale production scenarios. Since class II lanthionine synthetases utilize cofactors such as Zn^2+^ and Mg^2+^ in their catalysis (Garg et al., 2013; Yang and van der Donk, 2015; Repka et al., 2017), and the precursor peptide preRumA does not contain additional building blocks other than the canonical amino acids, cultivation in this medium was be able to facilitate formation of the PTMs in the peptide. The process was established by running parallel 24-microwell plate cultivations to optimize IPTG induction concentration and to establish a general overview of the strain behavior under different scenarios. Data obtained from these experiments were effortlessly transferred to the lab-scale high cell density bioreactor cultivation.

The rate of glucose release in the 24-microwell plate cultivations was controlled by altering the amount of glucoamylase in the EnPresso medium. Glucoamylase catalyzed the release of glucose from a soluble complex polysaccharide component of the medium in a concentration-dependent manner (Šiurkus et al., 2010; Glazyrina et al., 2012; Krause et al., 2016). Higher concentrations of the glucoamylase generated excess glucose in the medium. Report elsewhere shows that when high concentrations of IPTG is used to induce a recombinant gene, glucose uptake capacities of *E. coli* cells are severely reduced, and their abilities to respire optimally depreciates, leading to formation and accumulation of acetate (Neubauer et al., 2003). Acetate accumulation may explain why the pH of the plate cultures decreased with increasing concentration of glucoamylase. Overaccumulation of acetate may become toxic and slow cell growth, although *E. coli* may also oxidize the organic acid as an alternative carbon source (Roe et al., 2002; Neubauer et al., 2003). Nevertheless, to circumvent eventual acetate toxicity, feeding rate was controlled in the bioreactor cultivations by maintaining glucose concentration in the culture broth below 2 g L^–1^.

We took notice of the fact that the thickness of bands on SDS-PAGE representing target proteins extracted from end point samples obtained during the 24-microwell plate cultivations did not directly correlate with GFP fluorescence signals. Since the biosynthesis pathway requires the expression of both the precursor peptide and the modifying enzyme, we posited that it would be interesting to quantify product formation before and after the fluorescence signals began to decline, to ascertain expression quality. Therefore, samples were collected at different time points during the bioreactor cultivations and results from SDS-PAGE showed that IPTG induction of 10 h is enough to obtain good expression of both modifying enzyme and the substrate.

One limitation of the current study is that the precursor substrate is fused to a larger protein (GFP) that may interfere with the natural dynamics of the catalytic process. However, previous work indicated that the fusion of GFP to preRumA does not interfere with the activity of RumM on the precursor peptide. In fact, His6-RumM was able to interact and form complexes with the precursor peptide fused to the C-terminus of GFP (Ongey et al., 2018), indicating that the peptide is highly exposed to allow such interactions. Nevertheless, an alternative strategy is to use a different lanthipeptide that reproducibly expresses in *E. coli* without the need of fusion partner. Another limitation was the lack of an adequate method to quench the activity of RumM from the time the cells were collected throughout the process of extracting and analyzing preRumA. It is conceivable that the enzyme may have exhibited some activity during this time, thereby rendering our report on the modification kinetics inadequate. However, the fact that fully dehydrated preRumA was visibly present within 1 h of induction, but no ring was detected in the same sample may indicate otherwise. It was expected that if RumM was still actively catalyzing PTM formation during sample processing and measurements, thioether cross-bridges would have also been detected in this sample. The data reported herein qualitatively illustrates that it is possible to apply a combination of bioreactor fermentation and mass spectrometry to study *in vivo* kinetics of lanthipeptide modification, setting the basis for more rigorous investigation in that direction. Nevertheless, better approaches are needed to quench possible side reactions during sample processing to give a clearer view of the *in vivo* modification process. Additionally, a system that allows MS measurements in real time would be more useful in generating accurate quantitative data. We strongly believe that data from the optimized *in vivo* modification experiments would supply more information to enable the establishment of improved biotechnological systems to produce lanthipeptides.

## Conclusion

The data presented herein describes the production process of modified preRumA, established by applying consistent glucose-limited fed-batch cultivations from microwell- to bioreactor-scale. The model example illustrates that it is possible to produce extensively modified lanthipeptides in *E. coli* under standard fed-batch cultivation conditions without the requirements of additional cultivation media supplements. The heterologous strain demonstrated robustness judging from the two cultivations performed, while production yields in the range of 120–200 mg L^–1^ and some information about *in vivo* lanthionine synthesis by LanM enzymes were obtained. Results also suggest that *in vivo* catalysis by lanthionine-generating enzymes may be faster than what is observed in *in vitro* experiments. The dehydratase and cyclase reactions catalyzed by RumM is independent of each other and depending on the complexity of the ring architecture in the peptide, cultivation of heterologous *E. coli* hosts may be finished already 10 h after induction.

## Data Availability

All datasets generated for this study are included in the manuscript and/or the Supplementary Files.

## Author Contributions

EO and PN conceived the study. EO designed and analyzed the experiments, conducted all other experiments, and analyzed the data. LS, SW, and SR performed the bioreactor cultivation experiments. SR supervised the bioreactor experiments. EO and LS drafted the manuscript. EO, LS, and SW performed the mass spectrometric analyses. EO and LA did the data evaluation. PN supervised the study. SR and PN revised the manuscript. All authors read and approved the final manuscript.

## Conflict of Interest Statement

The authors declare that the research was conducted in the absence of any commercial or financial relationships that could be construed as a potential conflict of interest.
