# Supplementary material for: Bioprocess Development for Lantibiotic Ruminococcin-A Production in Escherichia coli and Kinetic Insights Into LanM Enzymes Catalysis
Source: Front Microbiol. 2019 Sep 13;10:2133. doi: 10.3389/fmicb.2019.02133 (PMC6753504; doi:10.3389/fmicb.2019.02133)
Supplement: Supplementary file 1 [file Data_Sheet_1.pdf]

Supplementary Material

Bioprocess development for lantibiotic ruminococcin-A production in *Escherichia coli* and kinetic insights into LanM enzymes catalysis

Elvis L. Ongey[1], Lara Santolin[1], Saskia Waldburger[1], Lorenz Adrian[2,3], Sebastian L. Riedel[1] and Peter Neubauer[1*]

*Correspondence: *Peter Neubauer*

E-mail: peter.neubauer@tu-berlin.de

***Correspondence address**

[1]Bioprocess Engineering

Institute of Biotechnology

Technische Universität Berlin

Ackerstraße 76, ACK24

D-13355 Berlin, Germany

Tel.: +493031472269

Fax: +493031427577.

1. Expression Vector

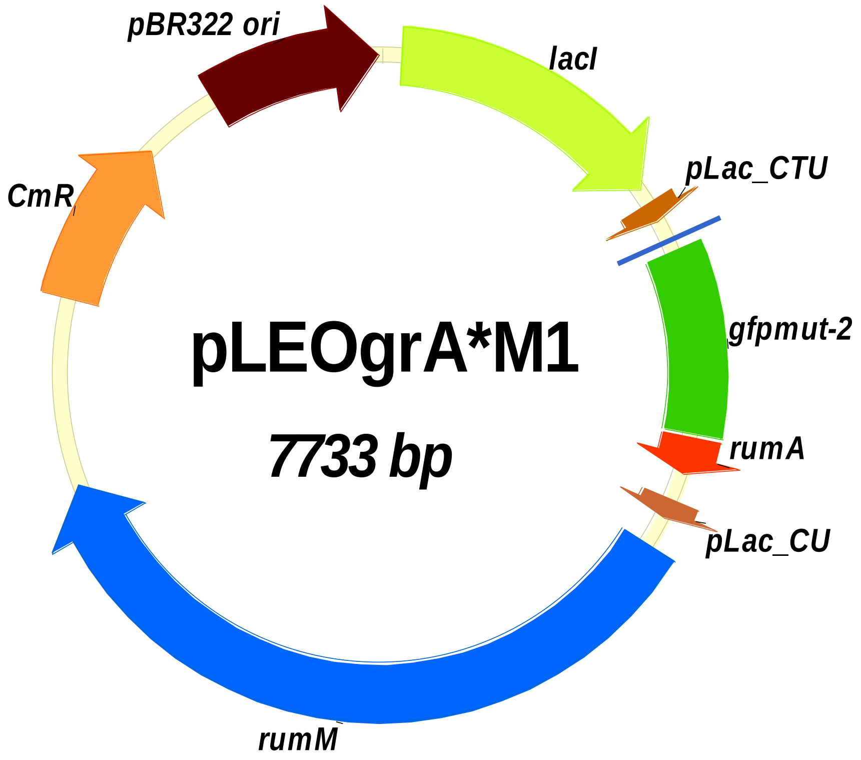

Figure S1 | Maps of polycistronic expression plasmid indicating all necessary features

2. 24 deep well plate experimental set-up

		1	2	3	4	5	6
A	Reagent A [U]	0.8	1.2	1.6	2	2.4	2.8
	IPTG [µM]	20	20	20	20	20	20
B	Reagent A [U]	0.8	1.2	1.6	2	2.4	2.8
	IPTG [µM]	50	50	50	50	50	50
C	Reagent A [U]	0.8	1.2	1.6	2	2.4	2.8
	IPTG [µM]	100	100	100	100	100	100
D	Reagent A [U]	0.8	1.2	1.6	2	2.4	2.8
	IPTG [µM]	500	500	500	500	500	500

Figure S2│ 24-well plate experimental design for screening optimal growth and production conditions in EnPresso B medium. The concentrations of reagent A (glucoamylase) and IPTG are indicated for each of the 24 wells.

3. Enpresso B® Preculture with PreSens System

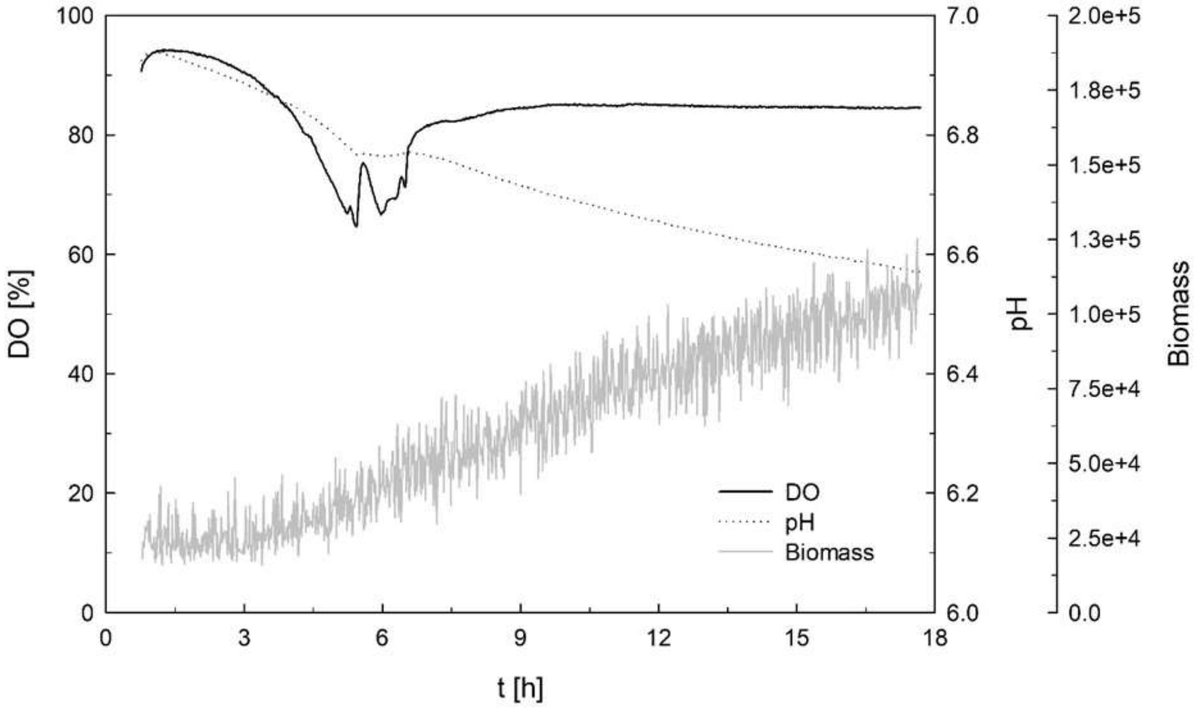

Figure S3 │ Overview of the Enpresso B preculture used for inoculation of the 1st reactor cultivation. *On-line* measured DO (%), pH and biomass (AU) are shown (PreSens).

4. Gel images

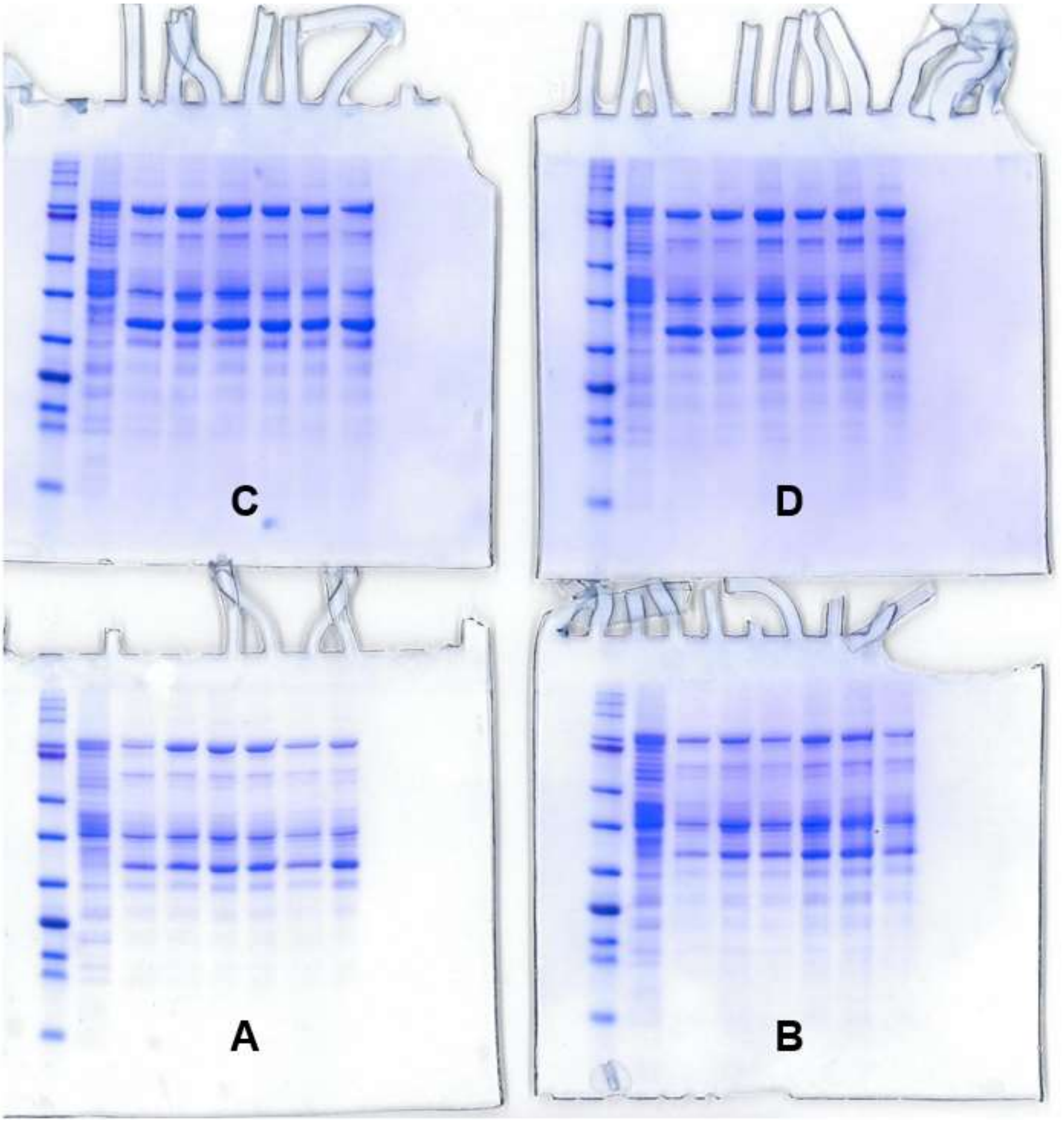

Figure S4│ Full gel images for expression optimization experiments performed in 24 deep well plates. His-tag spin column purified samples were loaded on the gel. A – D represents columns of the 24 deep well plates.

5. Bioreactor cultivations

1st Bioreactor

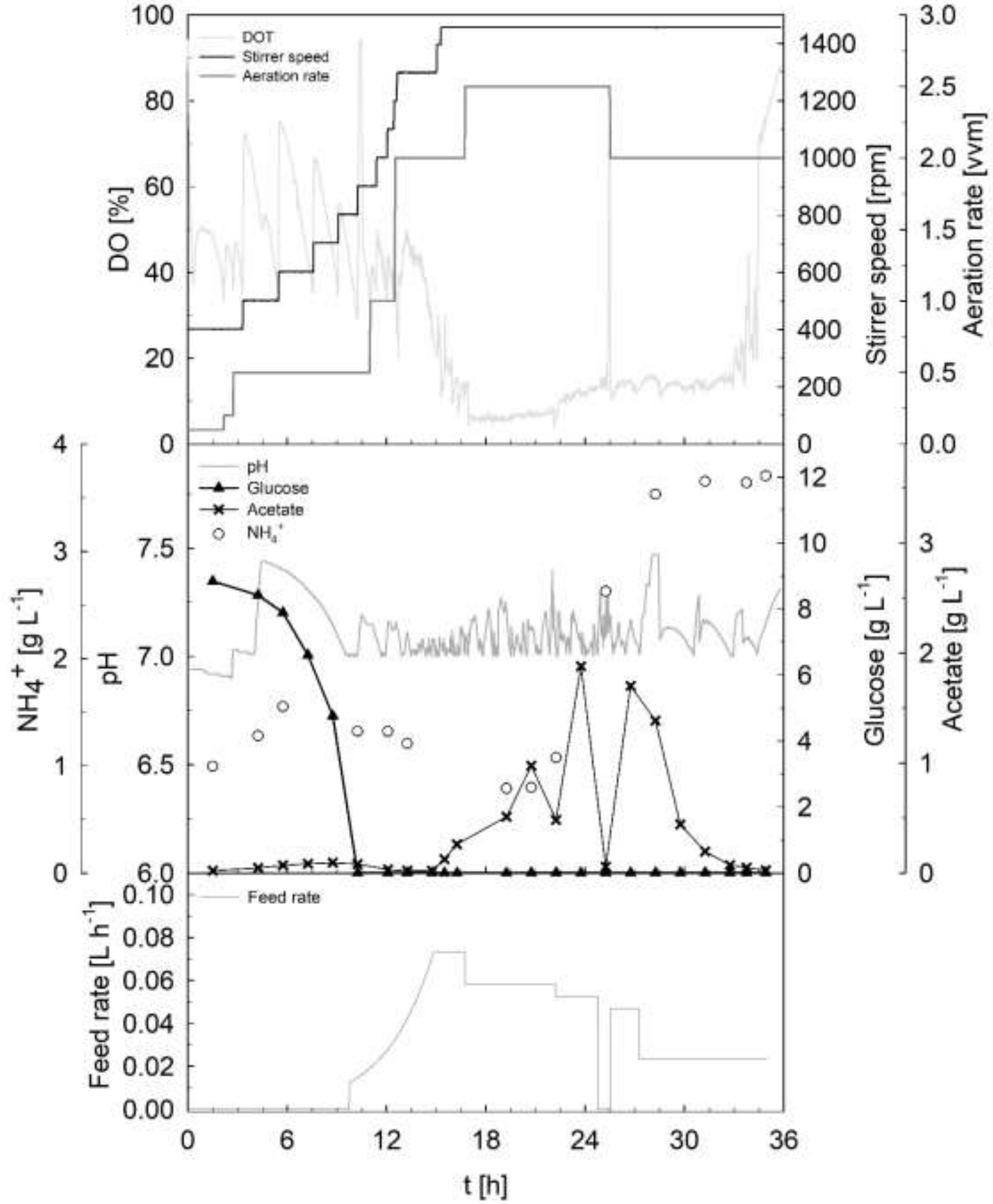

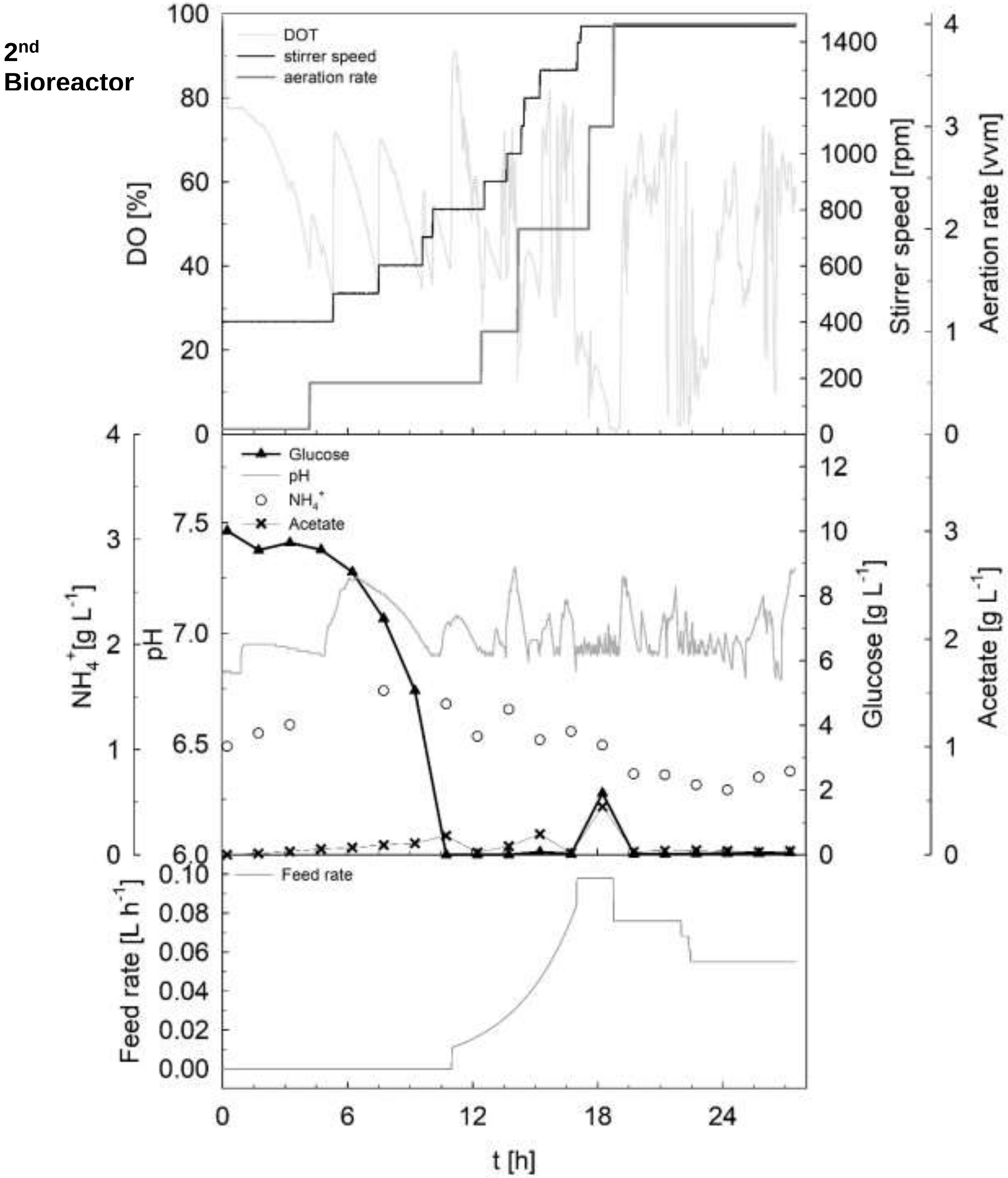

Figure S5│ Process parameters during bioreactor cultivations. *In-line* measurements of DO (%) and pH; applied stirring speed (rpm), aeration rate (vvm) and feeding rate (L h^{-1}) and *off-line* measurements of glucose (g L^{-1}), acetate (g L^{-1}) and NH_4^+(g L^{-1}) are shown.

1st Bioreactor Cultivation

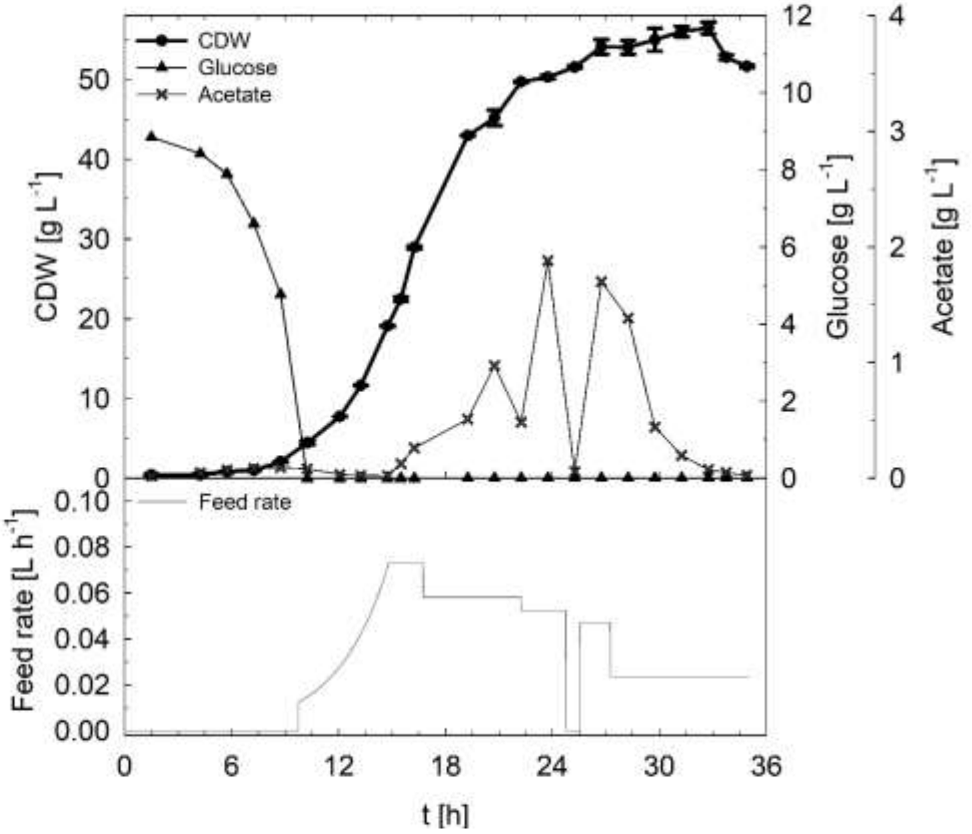

2nd Bioreactor

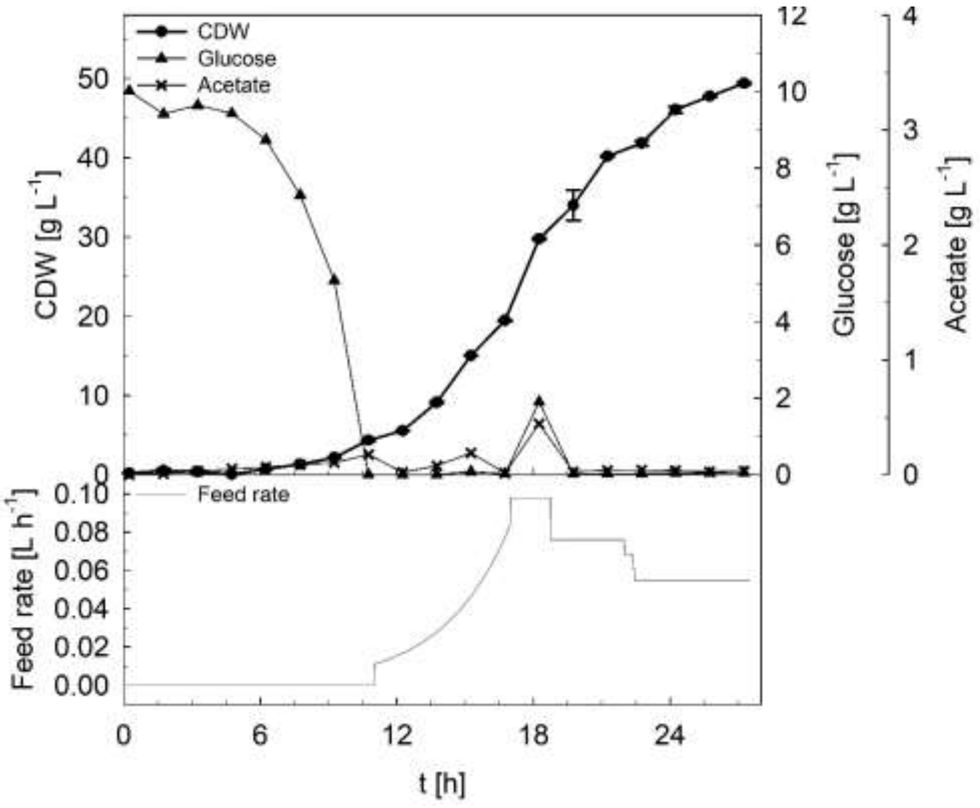

Figure S6│ Cultivation parameters during bioreactor cultivations. CDW (g L^{-1}), glucose (g L^{-1}), acetate (g L^{-1}) and feed rate (L h^{-1}) values are shown. Error bars indicate ± SD between duplicates.

1st Bioreactor Cultivation

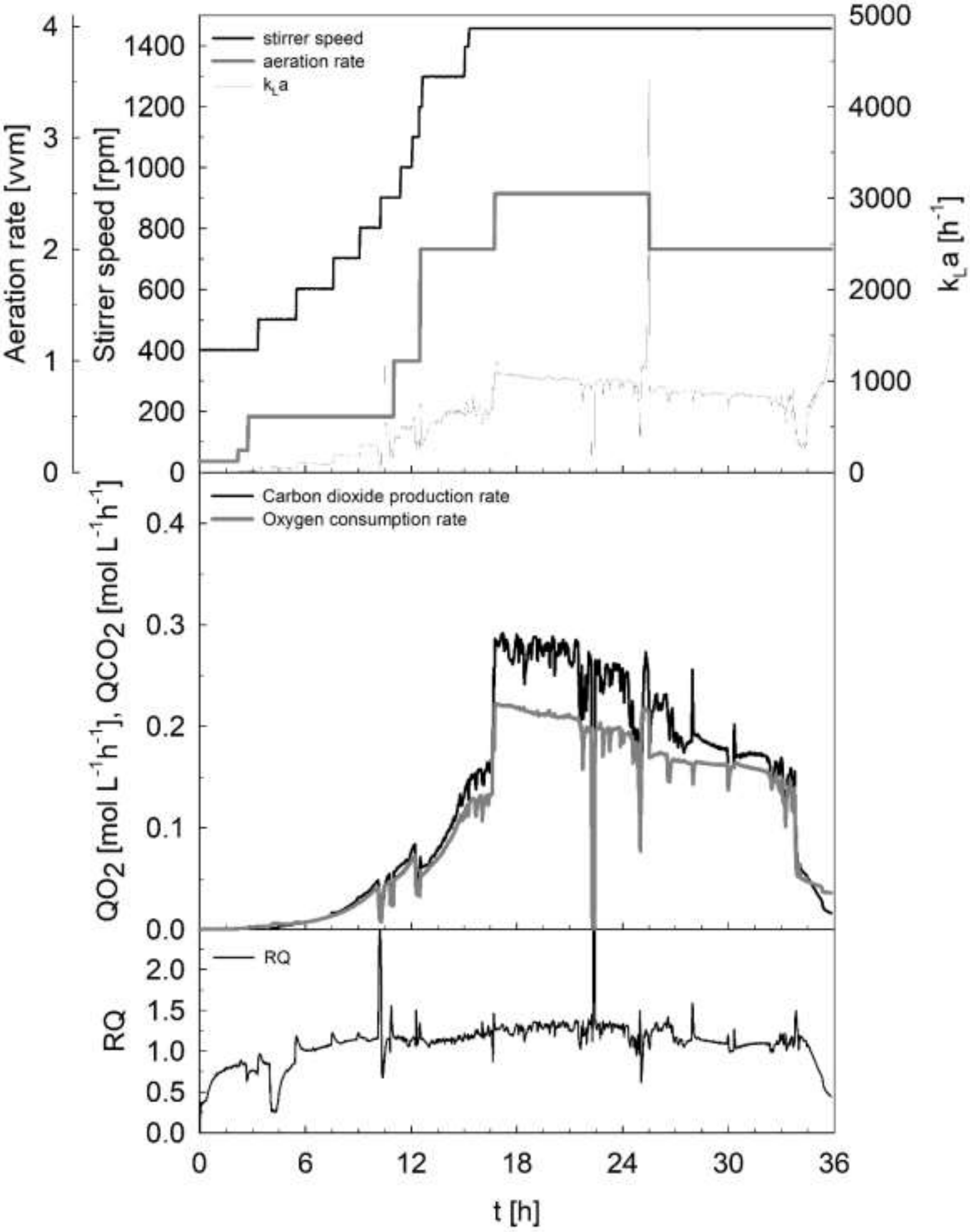

2nd Bioreactor Cultivation

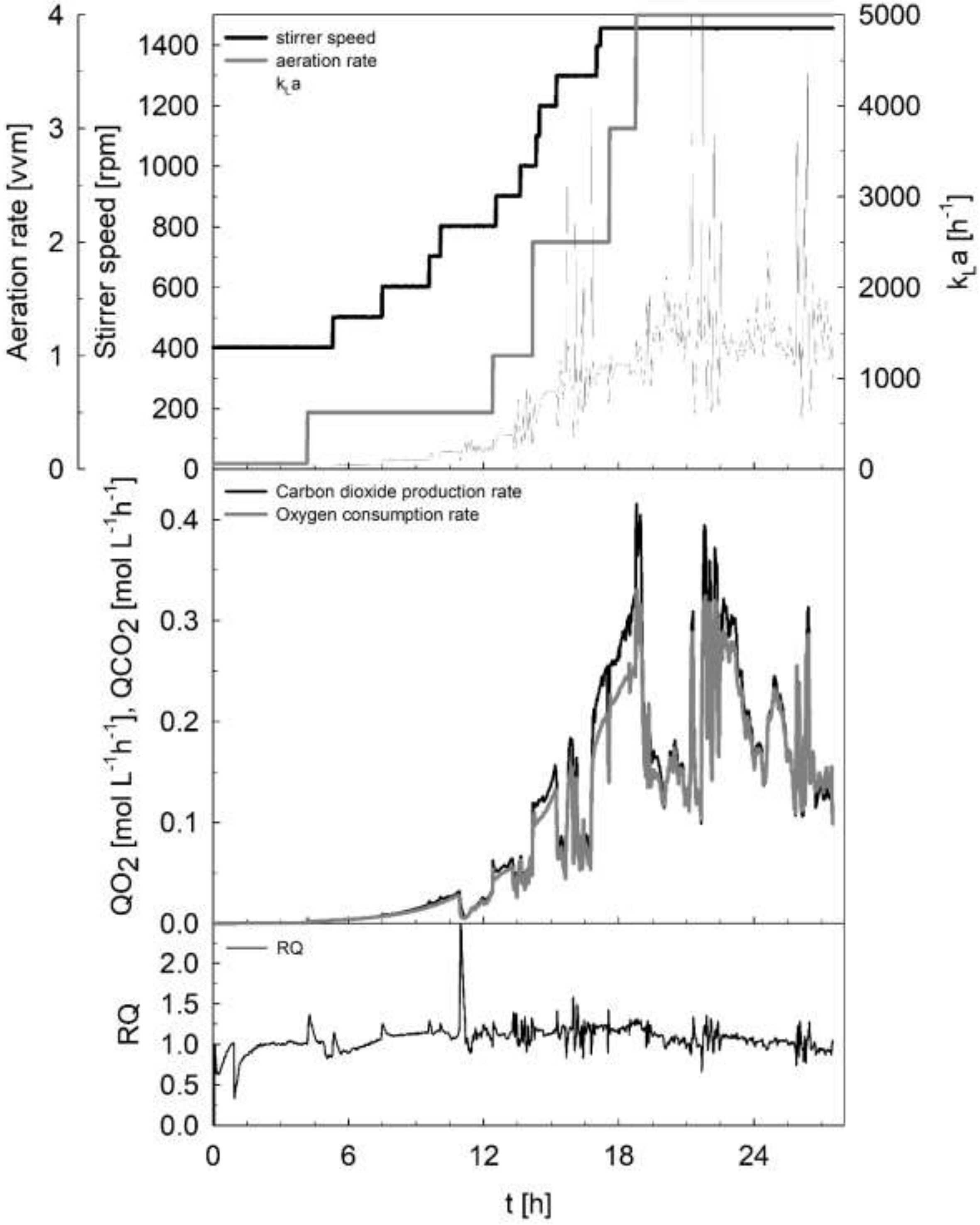

Figure S7 | Offgas analysis of bioreactor cultivations. Applied stirring speed (rpm) and aeration rate (vvm) and calculated volumetric mass-transfer coefficient (k_La h-[1]), oxygen uptake rate (QO_2 mol L^{-1} h^{-1}) carbon dioxide production rate (QCO_2 mol L^{-1} h^{-1}) and respiration coefficient (RQ) values are shown.

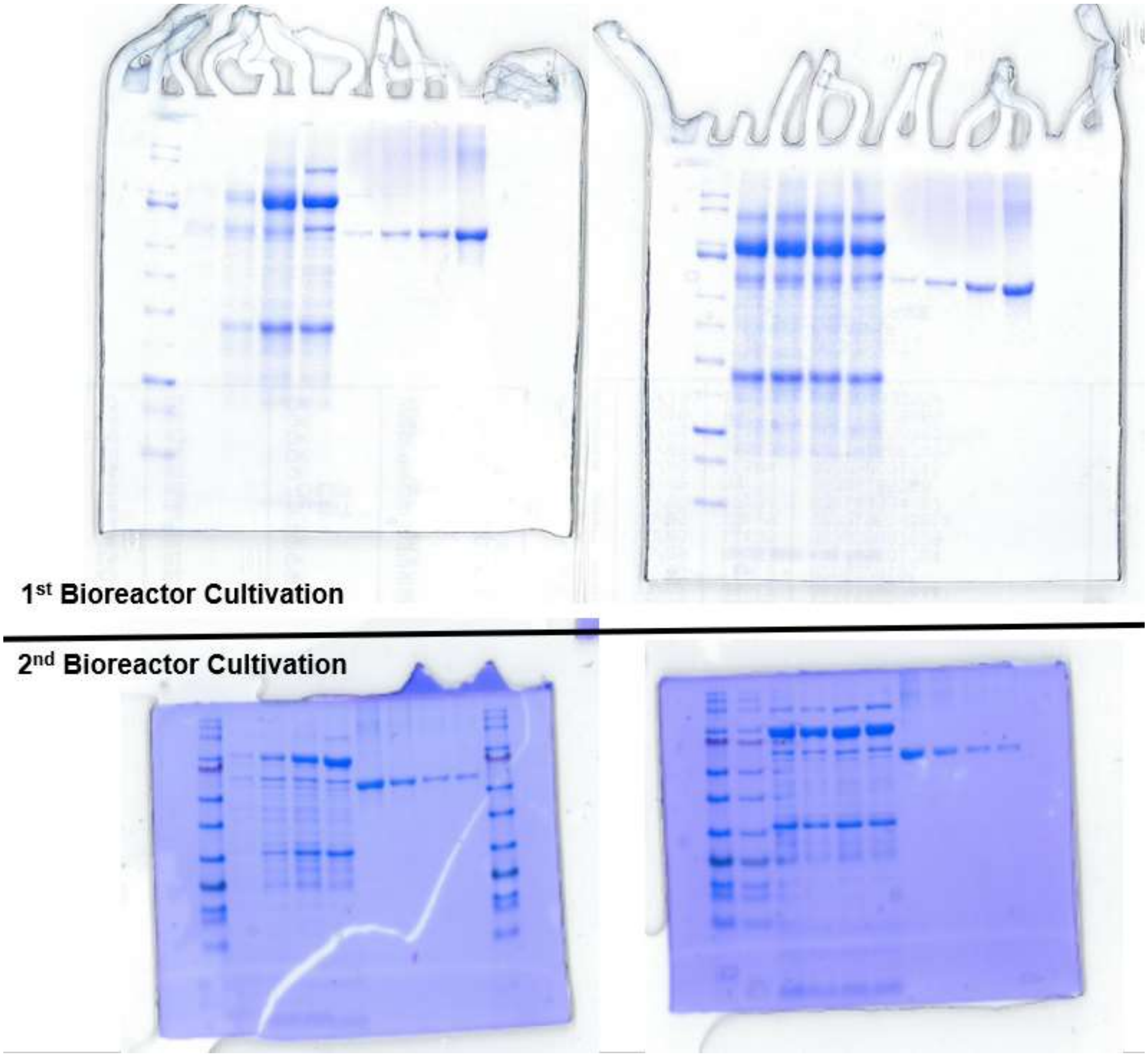

Figure S8 | Full gel images obtained from bioreactor cultivations. The areas of the bands in the gels were estimated using ImageJ and then compared against the BSA standards to obtain approximate quantities in mg l^{-1} which were subsequently plotted as shown in Figure 4B of the main text.

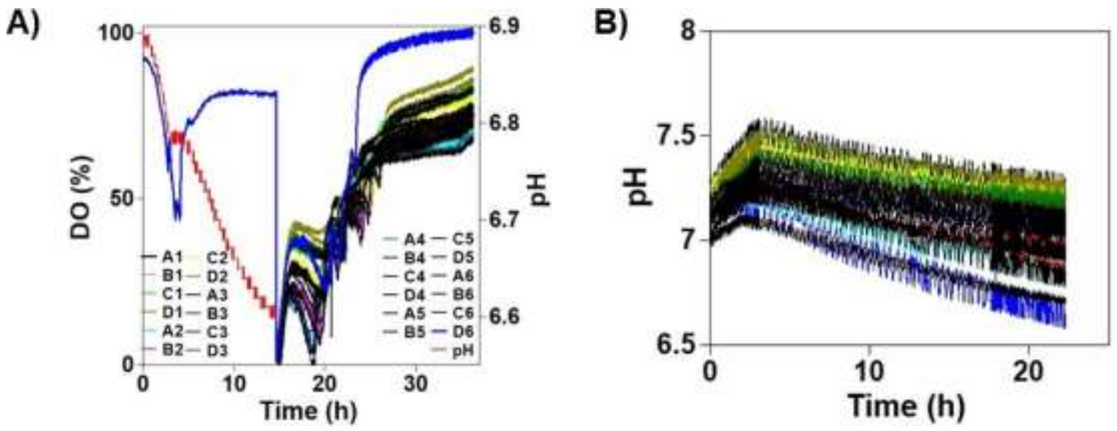

Figure S9│ Screening cultivation-relevant parameters of *E. coli* W3110 pLEO*grA*M1*. (A) DO of cultures before and after boosting/induction, and pH before induction. (B) pH of cultures after boosting/induction. pH of the culture before distribution into 24 microwell plate and induction is plotted in (A) and indicated by the red zigzag line.
